# Radiation treatment planning with embedded dose escalation

**DOI:** 10.1186/s13014-019-1348-3

**Published:** 2019-08-14

**Authors:** William T. Hrinivich, Todd R. McNutt, Jeffrey J. Meyer

**Affiliations:** Dept. of Radiation Oncology and Molecular Radiation Sciences, Johns Hopkins University School of Medicine, 401 N Broadway St. Weinberg Suite 1440, Baltimore, MD 21231 USA

**Keywords:** Stereotactic body radiotherapy, Intensity modulated radiotherapy, Dose escalation, Radiotherapy optimization

## Abstract

**Background:**

Heterogeneous target doses are a common by-product from attempts to improve normal tissue sparing in radiosurgery treatment planning. These regions of escalated dose within the target may increase tumor control probability (TCP). Purposely embedding hot spots within tumors during optimization may also increase the TCP. This study discusses and compares five optimization approaches that not only eliminate homogeneity constraints, but also maximize heterogeneity and internal dose escalation.

**Methods:**

Co-planar volumetric modulated arc therapy (VMAT) plans were produced for virtual spherical targets with 2–8 cm diameters, minimum target dose objectives of 25 Gy, and objectives to minimize normal tissue dose. Five other sets of plans were produced with additional target dose objectives: 1) minimum dose-volume histogram (DVH) objective on 10% of the target 2) minimum dose objective on a sub-structure within the target, and 3–5) minimum generalized equivalent uniform dose (gEUD) objectives assuming three different volume-effect parameters. Plans were normalized to provide equivalent maximum OAR dose and were compared in terms of target D0.1 cc, ratio of V12.5 Gy to PTV volume (R50%), monitor units per 5 Gy fraction (MU), and mean multi-leaf collimator (MLC) segment size. All planning approaches were also applied to a clinical patient dataset and compared.

**Results:**

Mean ± standard deviation metrics achievable using the baseline and experimental approaches 1–5) included D0.1 cc: 27.7 ± 0.8, 64.6 ± 10.5, 56.5 ± 10.3, 48.9 ± 5.7, 44.8 ± 5.0, and 37.4 ± 4.5 Gy. R50%: 4.64 ± 3.27, 5.15 ± 2.32, 4.83 ± 2.64, 4.42 ± 1.83, 4.45 ± 1.88, and 4.21 ± 1.75. MU: 795 ± 27, 1988 ± 222, 1766 ± 259, 1612 ± 112, 1524 ± 90, and 1362 ± 146. MLC segment size: 4.7 ± 1.6, 2.3 ± 0.7, 2.6 ± 0.8, 2.7 ± 0.7, 2.7 ± 0.8, and 2.8 ± 0.8 cm.

**Conclusions:**

The DVH-based approach provided the highest embedded doses for all target diameters and patient example with modest increases in R50%, achieved by decreasing MLC segment size while increasing MU. These results suggest that embedding doses > 220% of tumor margin dose is feasible, potentially improving TCP for solid tumors.

## Background

Dose prescription in radiation oncology is one of the most fundamental considerations during the treatment planning process. Prescribed doses for a given clinical scenario are based on experience, historical precedent, and the results of clinical studies, including phase I clinical trials [1]. For the most part, especially for common tumors treated in various contexts (adjuvant or definitive), dose-fractionation schemes are reasonably well established and commonly employed. A stated dose-fractionation scheme, however, tells an incomplete story. Meeting organ at risk (OAR) dose constraints may lead to various compromises in tumor coverage [2]. In addition, doses well above the prescription dose (hot spots) may be delivered to sub-volumes within the tumor [3].

When there are no critical normal tissues ensconced within a tumor mass, hot spots most likely will only increase tumor control probability (TCP), without anticipated worsening of toxicities, and can additionally aid in achieving steep dose gradients outside of the target [4–7]. Radiosurgery treatment plans are a prime example of this approach to treating cancer with radiation. Based in considerations of beam penumbra, radiosurgery plans often prescribe dose to very low isodose clouds (e.g., 50–80%) at the margins of tumors, leading to optimal gradient of dose outside of the target, and, simultaneously, extreme hot spots within the tumor [8, 9]. With intensity modulated radiotherapy (IMRT) planning, relaxing homogeneity constraints in the target (allowing hot spots, although not purposely planning for them) can also improve OAR sparing [7, 10]. Increasing the magnitude and spread of hot spots within tumors should improve the TCP from fundamental tenets of radiation biology. This may be especially true if there is heterogeneity of tumor cell radiosensitivity (relating, for example, to repair capabilities or hypoxia) throughout the tumor mass [5].

Thus, two radiation plans that are predicted to be isotoxic because they both meet normal tissue constraints may 1) not be equivalent with respect to the TCP and 2) in fact not be isotoxic, as our knowledge of normal tissue dose-fractionation effects is incomplete, and the plan that has the sharper gradient of dose may ultimately be associated with a lower risk of normal tissue complication. To improve the TCP, the planner can purposely “pack” or embed high dose within a tumor target as a planning objective during the IMRT optimization process. This approach can be considered a special/non-traditional simultaneous integrated boost (SIB) planning approach with focus on internal boosting *within* the gross tumor. Such a concept has been used previously in Gamma Knife treatment planning [11]. While intentionally embedding high doses in tumors using IMRT is possible, it may be achieved using multiple optimization approaches which may not be equally effective with respect to dose escalation internal to the tumor and dose gradient external to the tumor [12, 13]. To the authors’ knowledge there has not been a comparison of optimization approaches in terms of maximum embedded doses achievable while maintaining a given dose to the tumor margin and maintaining a high dose constraint outside of the target.

In this report, we consider five approaches to this problem- Approach 1 involves the definition of an embedded dose objective within the target, without specifying its physical location, using a dose-volume histogram (DVH) objective. Approach 2 involves the creation of a defined sub-volume within the target to be treated with an integrated/embedded boost. This approach is already in use in the clinic in various forms [13–15]. Approaches 3, 4 and 5 involve maximizing the generalized equivalent uniform dose (gEUD) in the target using a biological modelling-based objective function [16]. These gEUD approaches have been proposed in previous studies [12]. We compared these approaches by optimizing co-planar VMAT plans for a series of spherical targets with varying diameters and analyzing the resultant dose distributions in terms of embedded hot spots and gradient outside the target. We also analyze the machine delivery parameters of each plan to characterize the mechanism leading to differences in dose between approaches, and implemented all approaches in a single patient example to investigate clinical feasibility with true patient anatomy and dose constraints.

## Methods

### Phantom plan generation

#### Target and avoidance structures

**Fig. 1:**
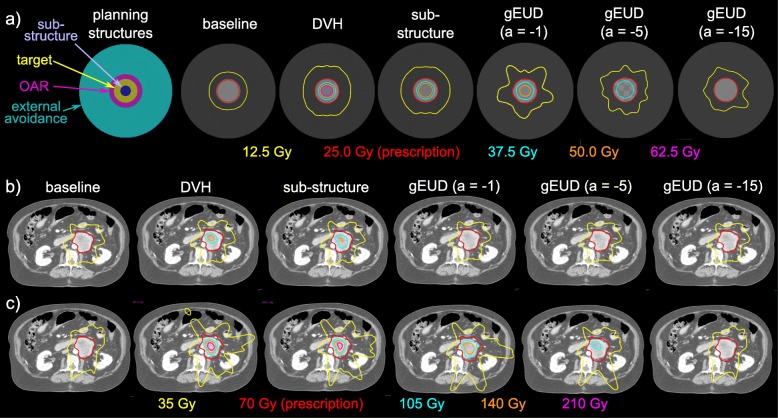
**a**) Axial views of the virtual CT, planning structures, and isodose lines for each planning approach for the 5 cm tumor diameter. **b**) Axial views of the patient plans optimized using each planning approach while meeting all OAR constraints and an R50% < 3.4. **c**) Axial views of the patient plans optimized using each approach while meeting all OAR constraints and relaxing the dose objectives limiting the R50%

Spherical targets with diameters varying from 2 cm to 8 cm in 1 cm increments were created and embedded in the center of a virtual cylindrical phantom with 20 cm diameter and uniform Hounsfield units of 0 using DICOMan software [17]. For each target size, a virtual OAR was created as a 1 cm thick spherical shell fully encompassing the target. External avoidance structures were created encompassing the phantom, excluding the target and OAR for each diameter. Axial views of the CT and planning structures are shown in Fig. 1.

#### VMAT optimization techniques

One baseline and five experimental optimization approaches were investigated in this study. Co-planar VMAT plans were produced using each optimization approach for each target diameter using Pinnacle 9.10 treatment planning software (Philips, Fitchburg WI). Plans consisted of two full co-planar arcs using the beam model for a VersaHD linear accelerator with an Agility MLC consisting of 160 5 mm wide leaves (Elekta, Stockholm SE). 6 MV energy with a flattening filter was used for all plans. Collimator angles of 15 degrees and 345 degrees were used for the first and second arc. All optimization approaches made use of the SmartArc optimization algorithm, which employs intensity modulation optimization to initialize arc segments followed by iterative gradient descent-based optimization of the machine delivery parameters such as leaf position and dose rate [18]. Differing dose objectives used in each planning approach modify the objective function minimized by the algorithm, which incorporates the sum of the squared differences between the specified and achieved dose objectives. These differences in the objective function lead to differences in the final dose distribution; however, characteristics of the iterative gradient descent-based algorithm will be consistent between approaches, such as susceptibility to local minima.

Optimization objectives for the experimental planning approaches were selected to maximize the dose embedded in the target while limiting toxicity by 1) respecting the maximum OAR dose constraint and 2) maximizing dose gradient (fall-off) beyond the target edge. Accordingly, optimization objectives intended to control OAR dose and dose gradient were kept constant for all plans as listed in the first 3 rows of Table 1. These objectives did not represent hard constraints during optimization, so did not guarantee that the maximum OAR dose and dose gradient remained constant between planning approaches. However, it was possible to rescale the dose (i.e. pick a prescription isodose line) that made all plans equivalent in terms of maximum OAR dose following optimization. Accordingly, all plans were normalized to provide an OAR D0.1 cc of 24.5 Gy. Maximum target doses and dose gradients could then be systematically compared between planning approaches to investigate the trade-off between these two plan features. An ideal optimization approach would enable significant dose escalation within the target (high dose embedment) while providing the same or similar dose gradient outside of the target.

**Table 1:** Plan optimization objectives

Optimization Approach	Structure	Metric	Value (Gy)	Weight
All (Max. OAR Dose Objective)	OAR	Max. Dose	24.5	5
All (Gradient Objective)	OAR	Max. D40%*	17.5	5
All (Gradient Objective)	External Contour Minus Target & OAR	Max. D10%**	7.5	5
Baseline	Target	Min. Dose	25	10
DVH	Target	Min. Dose	25	10
DVH	Target	Min. D10%**	80–100	0.1–10
Sub-Structure	Target	Min. Dose	25	10
Sub-Structure	Sub-Structure	Min. Dose	50–100	1–10
gEUD (a = −1)	Target	Min. gEUD (a = − 1)	100	10
gEUD (a = −5)	Target	Min. gEUD (a = − 5)	75	10
gEUD (a = − 15)	Target	Min. gEUD (a = − 15)	60–75	10

*dose to 40% of the structure, **dose to 10% of the structure

Objectives used to increase the embedded region of high dose varied between optimization approaches. For the set of baseline plans, a single *minimum* dose objective of 25 Gy was placed on the target with a weight of 10. Objectives intended to increase regions of high dose were selected for each target diameter based on the results of an analysis described in Section 2.2.2. Each optimization ran to convergence or a maximum of 60 iterations.

##### Approach 1

*Dose-Volume-Histogram (DVH)* The DVH approach involves placing a high minimum dose objective on 10% of the target volume (D10%), thereby allowing the region of escalated dose to be placed anywhere in the target by the plan optimizer. Although these dose objectives only apply to 10% of the target volume, the intention was to push the dose to a small portion of the target as high as possible. The resultant dose gradient between the embedded region of high dose and target edge leads to escalated doses delivered to the remainder of the target while respecting the surrounding OAR constraint. A minimum dose objective of 25 Gy was also placed on the original target to prevent regions of low dose outside of the sub-structure.

##### Approach 2

*Sub-Structure* This approach involves generating a sub-structure within the target by contracting the target uniformly to 10% of its original volume, and placing a high minimum dose objective on this structure. This sub-structure was thus placed at the center of the target. This technique allows the user to define the location of escalated dose to be at the center of the target, but also constrains the optimizer in terms of the physical location of escalated dose within the target. Similar to approach 1, a minimum dose objective of 25 Gy was placed on the original target to prevent regions of low dose within the target.

##### Approaches 3–5

*Generalized Equivalent Uniform Dose (gEUD)* gEUD is a metric intended to summarize a heterogeneous dose distribution delivered to a given structure based on the structure’s sensitivity to maximum and minimum dose [16]. gEUD is calculated based on a structure’s DVH using Eq. 1

1 $$ gEUD={\left(\sum \limits_{i=1}^N{\upsilon}_i{D}_i^a\right)}^{\frac{1}{a}} $$

where *N* is the number of elements in the structure, *v*_*i*_ is the volume of an element, *D*_*i*_ is the dose delivered to the element, and *a* is a unit-less volume-effect parameter. Higher values of *a* increase sensitivity to small regions of high dose and lower values of *a* increase sensitivity to small regions of low dose. Positive values of *a* are typically used for normal tissues, negative values of *a* are used for tumors.

The gEUD optimization approaches involve placing a high minimum gEUD objective on the target. In this study, we compared the gEUD approach using *a* values of − 1, − 5, and − 15 [12]. This optimization was performed using the Pinnacle Biological Evaluation tools, which enables the definition of gEUD-based optimization objectives. Since the gEUD is a function of the entire DVH, a high minimum gEUD objective pushes the optimizer toward increasing dose everywhere in the target. In this way, a separate minimum 25 Gy dose constraint was not found to be necessary for the targets when optimized using gEUD, and was omitted. Similar to the DVH approach, this approach allows the region of escalated dose to be placed anywhere in the target by the plan optimizer.

### Example patient plans

A patient with oligometastatic carcinoma previously treated to a site of para-aortic lymphadenopathy was selected for re-planning. The prescription dose was 70 Gy in 28 fractions to a PTV with volume of 181 cc. OAR constraints included maximum doses of 60 Gy and 70 Gy to the duodenum and aorta, which both overlapped the PTV [13, 19]. To satisfy these OAR constraints, a relaxed PTV coverage specification of V70Gy ≥85% was used for all plans. Baseline plans were first optimized to maximize PTV coverage while satisfying OAR dose constraints without additional objectives to increase or decrease maximum PTV dose. The baseline plans were then copied and used to initialize each of the experimental planning approaches. Dose objectives were iteratively modified for each planning approach to maintain PTV coverage ≥85%, satisfy OAR constraints, and maximize internal tumor dose. Two sets of plans were produced for this patient case: 1) while also preserving an R50% < 3.4 for all cases using ring structures and 2) relaxing the R50% objectives to allow greater internal tumor doses.

### Plan analysis

#### Dose metrics

Following optimization, all plans were exported as DICOM-RT structure set and dose files. The structure set and dose files were analyzed using an in-house application developed in C++ using the Insight Segmentation and Registration Toolkit (Kitware, Clifton Park NY), which rescaled dose to make OAR D0.1 cc equal to 24.5 Gy for each target size, and calculated relevant dose metrics for the target and surrounding phantom. Target D0.1 cc was calculated as an indication of the maximum dose embedded in the target. Target gEUD was calculated for all plans using *a* = − 1, *a* = − 5, and *a* = − 15 as an indication of radio-biologically equivalent dose assuming varying volume-effect parameters [16]. The fractional volume of the target receiving 37.5 Gy, or 150% of the intended marginal tumor dose (V150%) was also computed as an indication of the portion of the target receiving a substantially escalated dose [4]. The conformity index was calculated as the ratio of the 25 Gy isodose volume to the target volume as an indication of target coverage [20]. Finally, the ratio of the 12.5 Gy isodose volume (V12.5 Gy) to the target volume (R50%) was computed as a metric of dose gradient beyond the target edge [21].

#### Impact of D10% objective on maximum dose

To investigate the impact of the minimum D10% objective value on the resultant dose distribution when using the DVH and sub-structure optimization approaches, this parameter was varied from 25 Gy to 80 Gy in 5 Gy increments while keeping the normal tissue optimization constraints constant for the 5 cm target diameter. Each SmartArc optimization was run for 60 iterations [18]. Based on our experience with this simple phantom geometry, 60 iterations was sufficient to converge to a dose distribution that did not change with further iterations. The resultant dose distributions were analyzed in terms of dose to 0.1 cc of the target (D0.1 cc) as an indication of maximum achievable dose. This analysis was used to inform the dose objective choices for the remaining target sizes.

#### Machine delivery parameters

To investigate the mechanism leading to differences in dose distributions, the DICOM-RT plan files were also exported and analyzed in terms of monitor units (MU) and MLC leaf positions using an in-house Python script. Assuming that the 25 Gy prescription dose was delivered in 5 fractions, the number of MU required to deliver a single 5 Gy fraction was determined for each plan. The VMAT plans also incorporated modulated MLC positions. MLC positions were summarized for each plan by isolating the central 4 leaves (2 from each bank), and determining the average separation between the two opposing pairs of leaves over all control points (179 per beam). This mean MLC segment size was expressed as a distance in centimeters and also normalized by the corresponding target diameter and expressed as a fraction.

## Results

**Fig. 2:**
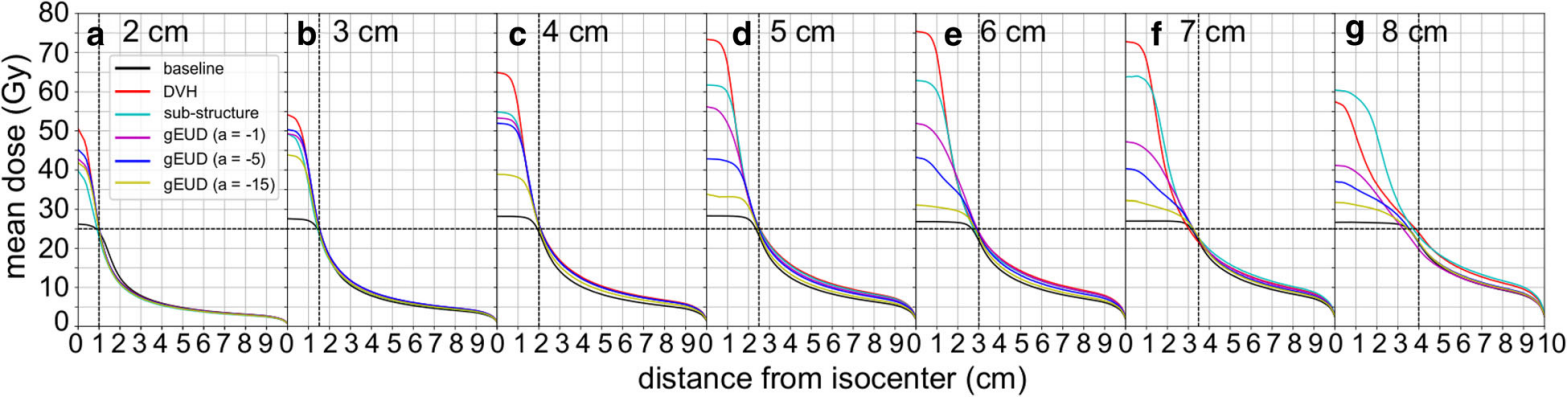
Mean dose in the axial plan versus distance from isocenter for each target diameter and planning approach. The horizontal dashed line indicates the prescription dose of 25 Gy and vertical dashed lines indicate target radii

**Figure 3:**
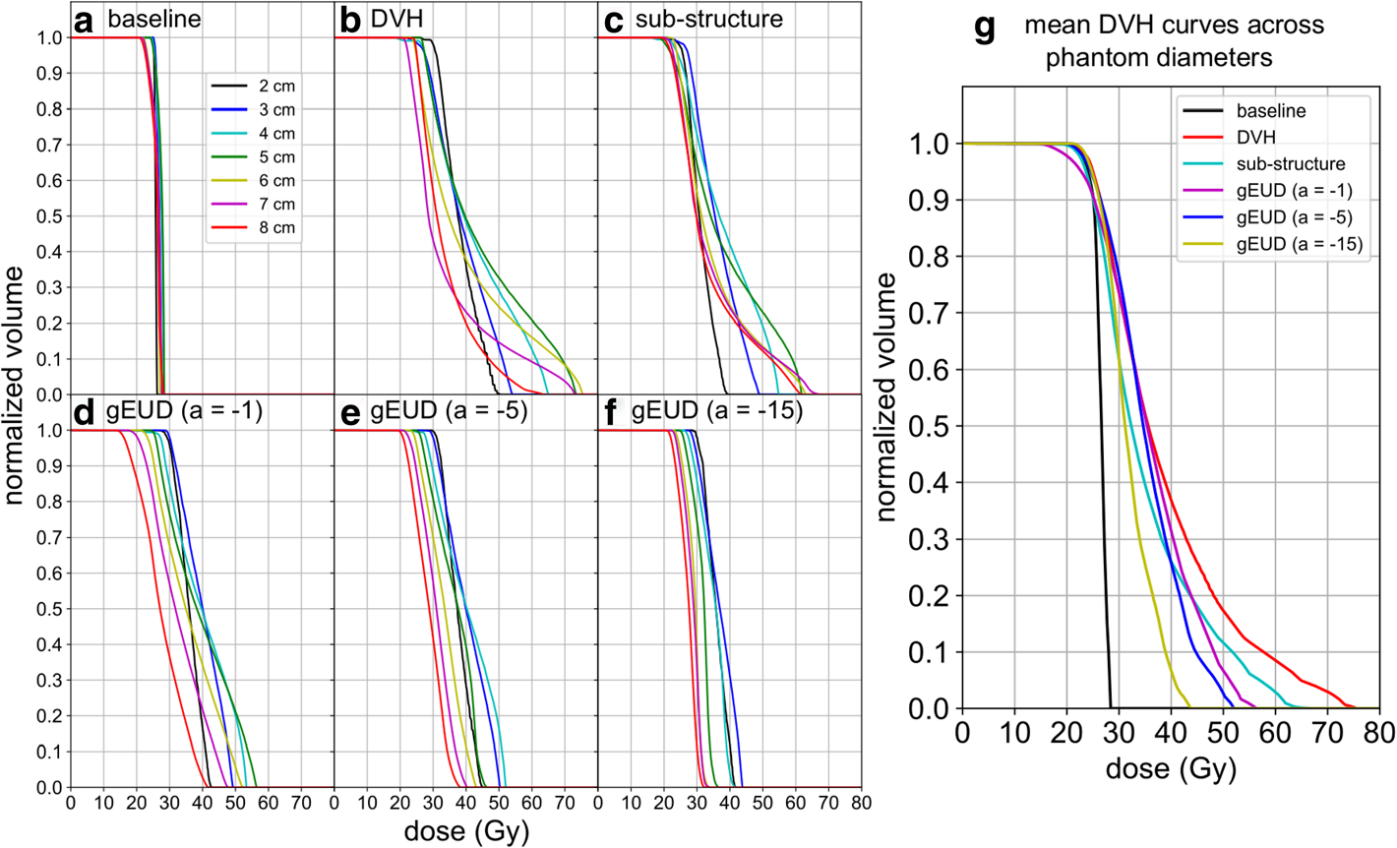
**a**-**f**) Cumulative DVH curves for each plan optimization approach and target diameter. **g**) Mean DVH curves for each optimization approach

Axial cross sections of the virtual CT and isodose lines from each planning approach are provided in Fig. 1a. The region of escalated dose within plans tended to be at the center of the target; however, for the DVH approach and 8 cm target diameter, the maximum dose was placed inferior to the center. Figure 2 provides corresponding mean dose profiles versus distance from isocenter for each planning approach, illustrating differences in central target dose and dose gradient. Fig. 3 shows cumulative DVH curves for all target diameters and optimization approaches investigated. Fig. 3 g plots the mean cumulative DVH curves across target diameters for each planning approach. Dose metric and machine parameter means and standard deviations are summarized for each optimization approach in Table 2.

**Table 2:** Mean ± standard deviation dose metrics for phantom and example patient plans

	Metric	Baseline	DVH	Sub-Structure	gEUD (a = − 1)	gEUD (a = − 5)	gEUD (a = − 15)
Phantoms	D0.1 cc (Gy)	27.7 ± 0.8	64.6 ± 10.5	56.5 ± 10.3	48.9 ± 5.7	44.8 ± 5.0	37.4 ± 4.5
	gEUD (a = − 1)(Gy)	26.4 ± 0.7	36.1 ± 3.4	33.0 ± 2.0	34.4 ± 4.6	34.4 ± 4.3	31.8 ± 3.6
	gEUD (a = − 5) (Gy)	26.3 ± 0.7	32.6 ± 3.1	29.9 ± 1.7	31.7 ± 4.9	32.7 ± 4.1	31.0 ± 3.6
	gEUD (a = −15) (Gy)	26.0 ± 0.9	28.6 ± 3.1	26.0 ± 2.1	28.0 ± 5.4	29.9 ± 3.9	29.6 ± 3.3
	V150%	0.00 ± 0.00	0.44 ± 0.13	0.32 ± 014	0.41 ± 0.18	0.36 ± 0.25	0.16 ± 0.21
	Conformity Index	1.02 ± 0.23	1.15 ± 0.32	1.00 ± 0.18	1.12 ± 0.39	1.18 ± 0.39	1.17 ± 0.36
	R50%	4.64 ± 3.27	5.15 ± 2.32	4.83 ± 2.64	4.42 ± 1.83	4.45 ± 1.88	4.21 ± 1.75
	Single Fraction MU	795 ± 27	1988 ± 222	1766 ± 259	1612 ± 112	1524 ± 90	1362 ± 146
	Mean Segment Size (cm)	4.72 ± 1.55	2.34 ± 0.66	2.61 ± 0.84	2.70 ± 0.69	2.73 ± 0.75	2.81 ± 0.79
	Mean Segment Size (normalized)	1.00 ± 016	0.50 ± 0.09	0.55 ± 0.10	0.59 ± 0.12	0.59 ± 0.11	0.61 ± 0.13
Patient - Constrained R50%	D0.1 cc (Gy)	99.6	160.2	143.2	97.2	100.2	98.3
	gEUD (a = − 1)(Gy)	76.3	81.8	83.3	77.1	76.9	76.4
	gEUD (a = − 5) (Gy)	73.5	76.3	76.6	74.0	73.6	73.5
	gEUD (a = − 15) (Gy)	57.2	59.4	59.5	56.6	56.1	56.6
	V150%	0.0	0.14	0.19	0.0	0.0	0.0
	Conformity Index	0.82	0.84	0.83	0.83	0.83	0.84
	R50%	3.37	3.37	3.29	3.01	3.15	3.15
	Single Fraction MU	834.6	1194.9	1160.2	878.5	862.5	856.5
	Mean Segment Size (cm)	4.03 ± 0.01	2.99 ± 0.03	2.95 ± 0.02	3.81 ± 0.04	3.90 ± 0.01	3.96 ± 0.01
Patient – Unconstrained R50%	D0.1 cc (Gy)	91.9	267.1	221.8	171.0	119.3	98.2
	gEUD (a = −1)(Gy)	79.5	90.2	90.3	98.2	84.9	77.2
	gEUD (a = −5) (Gy)	73.2	79.3	79.3	85.1	78.8	74.2
	gEUD (a = −15) (Gy)	61.5	61.6	66.1	64.2	55.4	59.5
	V150%	0.0	0.29	0.31	0.45	0.11	0.0
	Conformity Index	0.86	0.95	0.86	1.07	1.04	0.94
	R50%	3.94	5.20	5.04	5.70	4.79	4.02
	Single Fraction MU	688.9	1822.8	1682.7	1350.7	957.1	737.2
	Mean Segment Size (cm)	4.84 ± 0.03	2.08 ± 0.08	2.02 ± 0.03	2.55 ± 0.08	3.88 ± 0.03	4.65 ± 0.04

### Dose metrics

**Fig. 4:**
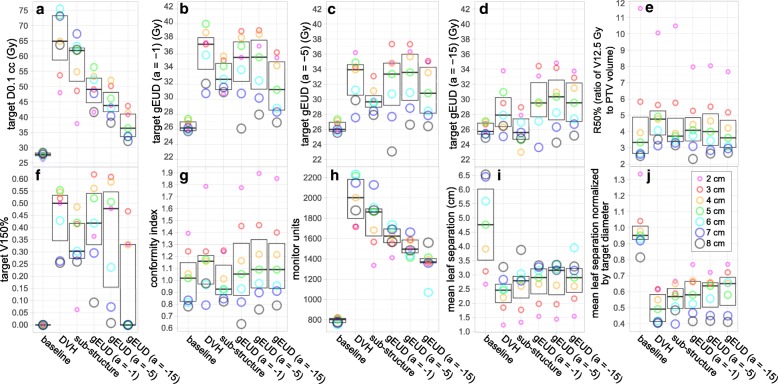
Boxplots of dose metrics and machine delivery parameters grouped by plan optimization approach. In each plot, the centerline indicates the median and the box indicates inter-quartile range. Values for each target diameter are indicated by dots

Figure 4 provides box plots of dose metrics for the six optimization approaches investigated. Values for each target diameter are indicated by dots. When considering target D0.1 cc, the DVH approach provided the highest mean value and highest values for each individual target size compared to all other optimization approaches. The DVH approach also provided the highest mean V150% of all optimization approaches. Alternatively, the gEUD approach with a = − 5 provided the highest mean target gEUD when analyzed using a = − 1, − 5, and − 15. The DVH approach and gEUD approaches provided similar conformity index values, which were higher than those provided by the sub-structure or baseline approaches, indicating a greater portion of the target receiving 25 Gy. Finally, the DVH approach led to the largest 50% isodose volumes, indicated by the highest R50% values. However, the average increase in R50% observed was only 11% above the average baseline values.

### Impact of D10% objective on maximum dose

**Fig. 5:**
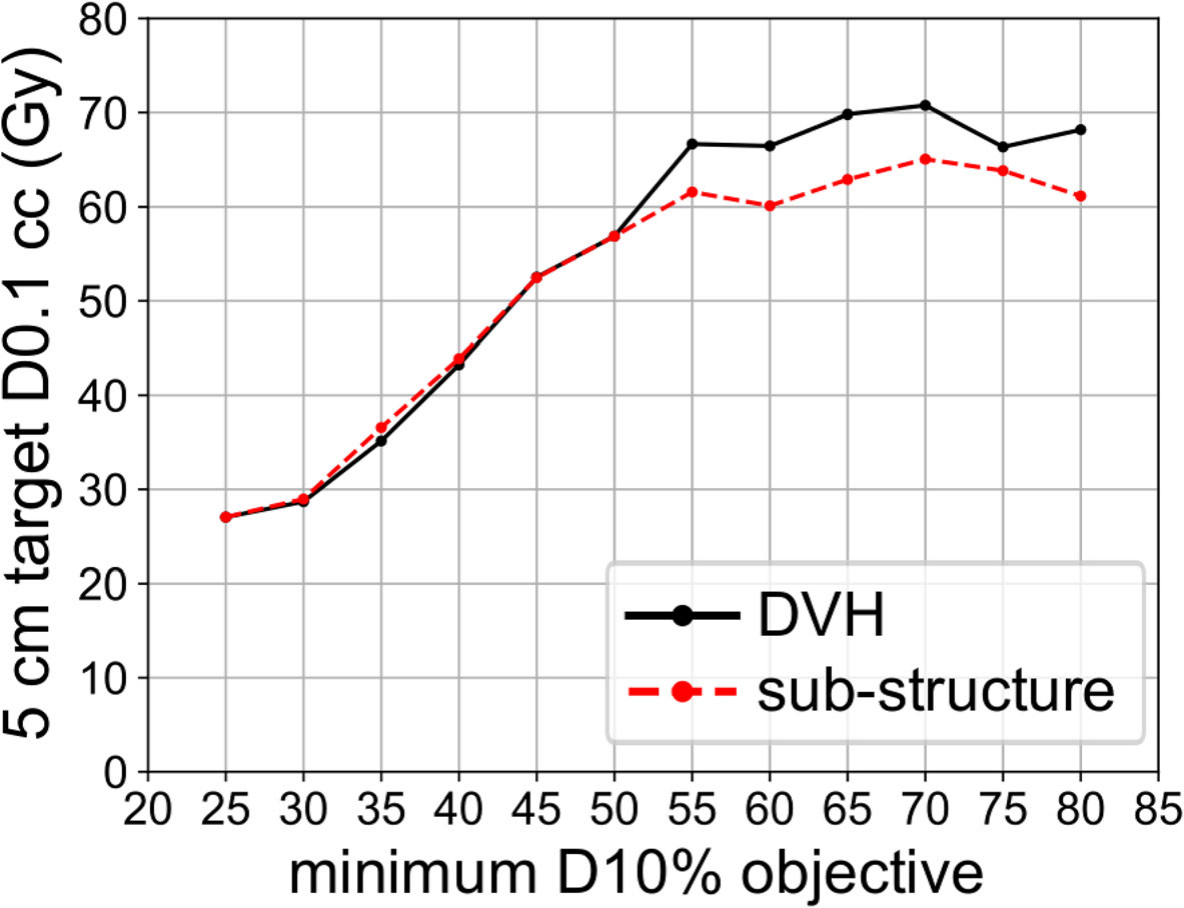
Target D0.1 cc versus minimum D10% objective for the 5 cm target diameter and DVH and sub-structure optimization approaches

Figure 5 displays target D0.1 cc versus minimum D10% constraint for the 5 cm target diameter and DVH and sub-structure optimization approaches. The target D0.1 cc depended on the minimum D10% objective for values of 25–55 Gy, beyond which the target D0.1 cc plateaued for both optimization approaches. The maximum achievable target D0.1 cc was higher for the DVH approach than sub-structure approach for all minimum D10% objective values ≥55 Gy.

Beyond an objective of 80 Gy, Pinnacle was not able to complete the plan optimization without reporting an error during the process for the 5 cm target size. Based on the observation of a plateau in D0.1 cc values before the 80 Gy limit was reached, we adopted an approach of selecting the highest minimum dose objective for each experimental planning approach and target diameter that allowed the optimization to complete without error. The achievable minimum dose objectives varied with target sizes as indicated in Table 1, and in some instances exceeded 80 Gy. In general, objectives had to be decreased for larger target diameters.

### Machine delivery parameters

Figure 4h provides boxplots of MUs for delivery of a single 5 Gy fraction for each optimization approach. All five experimental optimization approaches resulted in increased mean MUs by > 70% compared to the baseline approach. The DVH approach led to the highest mean MUs compared to all other optimization approaches. The trend in MUs between optimization approaches mimicked the trend in D0.1 cc. Figure 4i provides box plots of mean MLC leaf separation across all control points for each planning approach expressed in centimeters, and 4j provides box plots of the mean MLC separation normalized by target diameter. DVH approach led to the smallest MLC separation values of all optimization approaches. In general, plans with higher MUs were associated with decreased mean MLC leaf separation.

### Example patient plans

Axial cross sections of the example patient scan and isodose lines from each planning approach are provided in Fig. 1b for the plans with constrained R50%, and Fig. 1c for the plans with relaxed R50% objectives. Corresponding dose metrics are provided in the bottom two sub-sections of Table 2. The trends in dose metrics in the patient example mimicked the results obtained in the phantom plans. Specifically, the DVH approach provided the highest target D0.1cc compared to all other approaches, achieved by increasing MU and decreasing MLC separation, indicative of increased dose modulation. Target D0.1 cc of 229 and 382% of the prescription dose were achieved with and without an R50% constraint, respectively, while maintaining all other OAR constraints. However, relaxing the objectives minimizing the R50% in the final set of plans led to large increases in R50%.

## Discussion

This planning study compared five optimization approaches intended to embed high doses of radiation in tumors, thereby creating plans with extreme dose heterogeneity. While heterogeneous dose distributions are common in radiosurgery and stereotactic body radiotherapy (SBRT), this has typically been a byproduct of increasing the dose gradient at the tumor edge to improve normal tissue sparing, rather than purposeful embedding of hot spots within the tumor to improve TCP. This study demonstrates various optimization approaches to embed high doses within tumors, and demonstrates that there is minimal trade-off between increased tumor dose and decreased normal tissue dose with average increases in R50% ≤11%. Other approaches to embedded dose escalation beyond those studied here have also been reported, including the use of high-dose-rate (HDR) brachytherapy dose distributions as a guide for planning [22, 23]. It should be noted that embedded boosting differs from traditional SIB planning, which typically involves treating areas at risk for microscopic disease to a certain intermediate dose and areas of gross disease to a higher dose. Embedded boosting focuses on internal dose escalation *within* gross disease. The approaches investigated in this study maintained a minimum dose specification for the target indicated as the prescription dose, consistent with ICRU 83 level 2 dose reporting; however, central target doses significantly exceeded this dose level and could also be reported using maximum PTV dose or D2% to maintain an accurate record of the dose distribution [3].

Previous work has demonstrated that decreasing aperture sizes and increasing MUs provide increased target dose while maintaining dose outside of the target for radiosurgery [24, 25]. Tanyi et al. demonstrated that using a negative MLC margin of 1 mm resulted in superior TCP compared to zero or positive MLC margins for intracranial lesions treated with conformal arcs [24]. While increasing MUs is associated with increased OAR and integral dose, this effect is counter-acted by decreasing MLC separation which decreases beam overlap outside of the target. Similarly, our results show that the plan with the highest MUs and smallest mean MLC separation provided the highest embedded target dose. This plan was produced using the DVH-based optimization approach in both the phantoms and patient example. Alternatively, the gEUD-based optimization approach assuming a = − 5 provided the highest gEUD values of all optimization approaches. The relative impact of the high-dose regions provided by the DVH-based approach and the higher gEUD provided by the gEUD-based approach on TCP is unclear. The relative impact on TCP may depend on the distribution of tumor cell radio-sensitivity within the target [4, 5].

The DVH approach provided the highest embedded target doses compared to the other approaches for two potential reasons. 1) The DVH approach provides the optimizer with flexibility in terms of the size and shape of the embedded region of escalated dose, creating a larger viable solution space than the sub-volume approach leading to an improved solution. 2) The DVH approach directly emphasizes increased dose to a small sub-volume of the target, whereas the gEUD approaches emphasize increased dose to the entire target. In fact, the negative values of the gEUD volume-effect parameter *a* specifically emphasize the reduction of low-dose regions, rather than the creation of high-dose regions [16]. For these reasons, the DVH approach provided the most flexible and direct way to embed regions of high dose in targets. In this study, the DVH and sub-structure approaches were implemented using a 10% sub-volume of the PTV to enable initial comparison between approaches; however, this specific sub-volume value could be further optimized to improve results beyond those achieved in this study.

The minimum target dose objectives used for each of the five experimental planning approaches were selected to be as high as possible while still allowing the optimizer to either converge or complete 60 iterations without error. When objectives were selected that were higher than permissible, the treatment planning system would indicate that no further optimization could be performed after 10 iterations, returning a sub-optimal plan. This behavior was attributed to the instance of the SmartArc optimization algorithm employed in this study, which makes use of iterative gradient descent, and is therefore sensitive to initial conditions and is susceptible to local minima potentially leading to sub-optimal plans [18]. Optimizers available in other treatment planning systems may enable an increased range of dose objectives; however, we expect that the relative performance of the objective functions compared in this study to be applicable to other treatment planning systems making use of similar gradient descent-based optimization.

We also performed a limited investigation of the impact of the minimum D10% objective on the maximum achievable target dose for the DVH and sub-structure optimization approaches for the 5 cm target diameter, and found that increasing objective values > 220% of the desired tumor margin dose had little impact on the resultant region of escalated dose. We were able to use minimum D10% objectives > 220% of the tumor margin dose for all target diameters when using the DVH approach, and for target diameters ≤5 cm when using the sub-structure approach. We were able to use minimum D10% objectives of 200% of the desired tumor margin dose for the 6, 7, and 8 cm target diameters using the sub-structure approach. A minimum D10% objective of 200% of the desired tumor margin dose may be a pragmatic starting point when using the DVH or sub-structure approach. Although we have not yet performed a similar analysis for the gEUD optimization approaches, we were able to use minimum gEUD objectives > 220% of the tumor margin dose for all target diameters, which we expect to be within the plateau region of resultant target D0.1 cc.

The oncologic advantages of embedded hot spots within a tumor, and the relationship to the magnitude of the hot spots, are uncertain. Hot spots emerge naturally from treatments such as Gamma Knife radiosurgery and interstitial brachytherapy. Modeling studies have shown the ability of intra-tumoral boosts to increase tumor control probability [4–6]. Embedded hot spots would likely be especially beneficial if tumor cell radioresistance is heterogeneously distributed throughout the tumor [5]. A variety of clinical studies have investigated the relationship of peripheral tumor dose as well as internal hot spots to tumor control, with some reports showing an association between internal dose escalation and better tumor control outcomes [26, 27]. Ideally the areas of a tumor which contain the most resistant clonogens could be identified and selected for specified internal boost, and there is much interest in the use of imaging studies to identify tumor sub-volumes for boosting, but such information is often lacking from available imaging studies, and may in fact be fluid depending on, for example, oxygenation patterns during a radiation treatment course [28, 29]. As our knowledge of the impact of functional imaging studies on guiding embedded dose escalation evolves, our perspective is the following: for selected tumors which lack critical normal structures interspersed within the tumor, and for a given dose prescribed to the margin of the tumor, embedding high doses (beyond the tumor margin dose) within the tumor is likely to be beneficial, relative to conventional planning (without embedded hot spots), so long as the dose gradients outside of the tumor are acceptable (close or equal to the conventional plan). In this report, we have compared several approaches that can achieve this goal.

This planning study involved a simplified virtual phantom, targets, and OARs to compare optimization approaches in terms of the resultant dose distributions for varying target sizes. The impact of patient heterogeneity, abnormal target shapes, and overlap between PTV and OARs will lead to differences in the maximum achievable doses from those found in this study. Furthermore, we limited our investigation to a 6 MV beam with a flattening filter, commonly used for VMAT. Flattening filter free (FFF) beams may enable further increases in embedded target dose by providing a sharper penumbra and naturally peaked dose profile, as well as shorter delivery times for hypo-fractionated treatments [30]. Other non-coplanar treatment configurations such as 4π SBRT [31] or CyberKnife [32] may also enable further increases in central tumor dose and improved gradients by reducing beam overlap outside of the target, but comparison of these approaches was beyond the scope of this study. Finally, the results of this study do not take any geometric uncertainty into consideration, pre-supposing that the target is entirely solid tumor, without motion, allowing and motivating increases in dose within the target. These approaches would not be appropriate for targets with critical structures interspersed within the target, or targets in regions with large setup uncertainties or internal motion.

## Conclusions

The results of this virtual planning study suggest that a minimum D10% objective is an effective way to embed a high dose in a tumor, providing maximum internal doses of > 220% of tumor margin dose for targets 4–8 cm in size while preserving dose constraints to abutting OARs, achieved by increasing MUs and MLC modulation. The embedded hot spots lead to small increases in R50%, potentially leading to improved TCP while maintaining normal tissue toxicity for appropriately selected solid tumors.

## Data Availability

The datasets used and/or analysed during the current study are available from the corresponding author on reasonable request.

## Abbreviations

D0.1 cc: Dose to 0.1 cc of a structure based on a cumulative DVH
D10%: Dose to 10% of a structure based on a cumulative DVH
DVH: Dose volume histogram
gEUD: Generalized equivalent uniform dose
HDR: High dose rate
IMRT: Intensity modulated radiotherapy
MLC: Multi-leaf collimator
MU: Monitor units
OAR: Organ at risk
PTV: Planning target volume
R50%: Ratio of the volume of tissue receiving at least 50% of the prescription dose to the volume of the PTV
SIB: Simultaneous integrated boost
TCP: Tumor control probability
V12.5 Gy: Fractional volume of a structure receiving at least 12.5 Gy
V150%: Fractional volume of a structure receiving at least 150% of the prescription dose
VMAT: Volumetric modulated arc therapy
*a*: gEUD volume effect parameter
